# Awareness About Thalassemia Among the Parents of Thalassemic Children in Balochistan: A Cross‐Sectional Study

**DOI:** 10.1002/hsr2.70715

**Published:** 2025-04-18

**Authors:** Zarnum Gul, Mehtab Malik, Uzzi Uzair, Aiman Baloch, Quang Dai La, Noman Sadiq, David F. Lo

**Affiliations:** ^1^ Mekran Medical College Turbat Balochistan Pakistan; ^2^ The Innovative STEMagazine 501(c)(3) College Station Texas USA; ^3^ College View High School College Station Texas USA; ^4^ School of Arts and Sciences Rutgers University New Brunswick New Jersey USA

**Keywords:** awareness, Balochistan, β‐thalassemia, BTM, Pakistan

## Abstract

**Background and Aims:**

Thalassemia major is the most common genetic disorder, affecting 60,000 individuals annually worldwide. It involves reduced or absent synthesis of hemoglobin chains, leading to severe anemia. Awareness of its transmission, diagnosis, and prevention is crucial in reducing incidence, especially in high‐risk families. This study assessed parental awareness of thalassemia in Kech, Balochistan.

**Methods:**

A cross‐sectional study was conducted at Kech Thalassemia Care Center, Turbat, Balochistan, from May to October 2023, involving 190 parents of children diagnosed with β‐thalassemia major. Participants were selected using a convenience‐purposive sampling method. A structured questionnaire was developed, piloted, and used for data collection after obtaining informed consent. Ethical approval was granted by Mekran Medical College Turbat. Data were analyzed using SPSS 20. Bayesian linear regression analysis was applied to assess associations.

**Results:**

The mean ages of fathers and mothers were 38.18 ± 9.78 and 32.28 ± 7.80, respectively. Most participants (55.3%) lived in rural areas; 63.2% of mothers were illiterate, while 62.6% of fathers were educated. Only 13.7% recognized thalassemia as an inherited disease, and 91.6% were unaware of premarital and antenatal screening. Blood transfusion was seen as the only treatment by 81.1%, and 50% acknowledged the role of consanguinity. Awareness was poor in 48.9% and good in only 4.7% consanguinity and residency showed a significant association with awareness.

**Conclusion:**

Parental awareness of thalassemia was inadequate. residency and consanguinity were significantly associated with awareness, highlighting the need for targeted educational interventions.

**Abbreviations**
CAT: catalase
CI: confidence interval
COPD: chronic pulmonary lung disease
FEV1: forced expiratory volume in 1 s
FVC: forced vital capacity
IFN‐γ: interferon‐gamma
IL‐10: interleukin‐10
MDA: malondialdehyde
MMEF: mid‐maximum expiratory flow
NF‐κB: nuclear factor‐kappa B
PEF: peak expiratory flow
PFT: pulmonary function test
PRISMA: Preferred Reporting Items for Systematic Reviews and Meta‐Analyses
RCT: randomized clinical trial
RoB: risk of bias
SD: standard deviation
SE: standard error
SOD: superoxide dismutase
TNF‐α: tumor necrosis factor‐alpha
WMD: weighted mean difference

## 1 Introduction

Thalassemia is a genetic disease with diminished formation or absence of ⍺ or β chains of normal hemoglobin. The disease's transmission pattern is autosomal recessive, and depending on the type of globin chain affected by the mutation, the disease can be classified as ⍺‐ and β‐thalassemia [1]. Based on severity, β‐thalassemia has three types: The most severe form of the disease is known as thalassemia major, in which the patient requires lifelong blood transfusion; thalassemia intermediate has mild anemia; and asymptomatic disease with carrier state is known as thalassemia minor. Inadequate hemoglobin leads to chronic hemolytic anemia and presents with pallor, failure to thrive, and iron overload in the body [2].

Thalassemia major is the most frequently occurring disease, affecting 60,000 individuals annually worldwide [3, 4]. Recent epidemiological data suggest an increasing global burden, particularly in regions with high consanguinity rates. The genes for β‐thalassemia are present in about 3% of the world's population, and 60,000 new cases arrive every year. Regions such as Central Asia, Transcaucasia, the Far East, the Indian subcontinent, and the Mediterranean exhibit a higher prevalence of thalassemia [4]. In Pakistan, the situation is also concerning with an estimated 7 to 10 million people from diverse racial groups suffering from this disorder while the carrier rate in Pakistan is 5%–8%. The average life span of β‐thalassemia patients in Pakistan is approximately 10 years and an estimated 90,000 and 100,000 documented cases of the disorder are reported across the country. Reasons for the increasing frequency of disease reported by studies are poor knowledge, low‐income state, and marriages in common ethnic populations and close relatives [5]. The prevalence of hemoglobinopathies stated in northern Balochistan is 0.79%. The most frequent hemoglobinopathy among them was β‐thalassemia major (29.4%), β‐thalassemia heterozygous disease (28.3%), and sickle cell anemia (28%) [6]. In the province of Balochistan, there are approximately 2000 children living with β‐thalassemia major [7]. A recent study by Azmatullah et al. assessed the prevalence of congenital anomalies in Balochistan, identifying thalassemia major as the most common disorder among the blood‐heart category [8]. This underscores the significant impact of thalassemia in the region and the urgent need for targeted interventions. Despite these alarming figures, research on awareness levels among affected families in Balochistan remains limited [7].

Consanguineous marriage is the major reason leading to the high incidence of disease in Pakistan. Thalassemia major increases the financial burden on affected families because management modalities for thalassemia major include frequent blood transfusion, iron chelating medicines to treat iron overload, and management of problems like endocrine disorders, cardiac problems, Hepatitis B and C, osteoporosis, and HIV infection occurring by repeated blood transfusion. The only permanent cure for thalassemia major is bone marrow transplantation, but the majority of families cannot afford it [9]. Although treatment of thalassemia is not easily accessible, the only way to decrease its burden is disease prevention. Executing carrier screening programs, genetic counseling of couples, and prenatal diagnosis are the most successful interventions to reduce the incidence of disease. Education is one of the preventive strategies applied internationally. Information about the mode of inheritance, preventive measures, and presenting complaints of thalassemia should be given through an easy and comprehensive medium [2]. Many studies reported that awareness about thalassemia even among the affected people and their families in Pakistan is insufficient. Parents lack knowledge that it's a genetic disorder; they don't know about the mode of inheritance of the disease, and they don't show good attitudes and efforts to prevent it, that is, antenatal screening and abortion of the diseased embryo. Illiteracy, insufficient awareness initiatives, incompetent health services, a poor economy, and spiritual and societal norms are reasons behind the increasing burden of the disease in the country [10]. Thalassemia is a genetic disorder; therefore, it can only be managed by taking preventive measures. Educating the general population and improving the understanding and perception of affected families regarding the disease is an influential approach to averting thalassemia and decreasing the incidence and fatality of the disease [11]. Given these gaps, this study aims to assess the awareness levels of parents of thalassemia major patients in Kech, Balochistan, providing insights to inform targeted public health interventions in the region. Furthermore, the data collection of this paper was done concurrently with another study regarding the family planning practices among the parents of beta‐thalassemia patients [12].

## 2 Methodology

A cross‐sectional study was conducted in Kech Thalassemia Care Center in Turbat Balochistan among the participants whose children were affected by BTM from May 2023 to October 2023. The study included 190 parents of thalassemia major patients registered in the Kech thalassemia care center. There are 540 registered families in this center. The sample size of 225 was calculated using openEpi with a 95% confidence level. However, due to participant availability and consent constraints, the final sample size was 190. Convenience purposive sampling was used to select the parents. This method was chosen due to the logistical challenges of random sampling in a healthcare setting, ensuring the inclusion of parents who actively engaged with the center and were more likely to provide informed responses. Parents of thalassemia‐affected children visiting thalassemia centers on scheduled days and agreeing to participate were included in the study. In contrast, those who refused to give consent and parents of critically ill patients were excluded from the study for ethical reasons.

For data collection, 1 or 2 days a week, the thalassemia center was scheduled to be visited. The data was collected by a self‐made questionnaire, and before the study, written informed consent was taken from all the participants. The questionnaire has two sections. The first section includes Sociodemographic Characteristics, that is, name, age, address, ethnicity, religion, family income, educational level, caste, monthly income, total number of kids, and number of BTM‐suffering kids. The second section contained questions about thalassemia, its mode of transmission, premarital and prenatal screening, and treatment options to assess the knowledge of parents about thalassemia major. The questionnaire was checked and modified after a brief pilot study in the same study setting to make sure the questions were valid and comprehensive for the study population. Additionally, the study accounted for potential confounders such as access to healthcare and prior health education by including questions on participants' previous exposure to thalassemia‐related information and their healthcare‐seeking behaviors.

Ethical approval was obtained from the ethical committee of the Mekran Medical College Turbat under ref no. MMC/ERC:/01/2/2023. The statistical analysis was performed on SPSS version 20.

## 3 Results

### 3.1 Sociodemographic Details

In this study, a total of 190 diagnosed cases of thalassemia in children and their parents were enrolled. The age span of fathers was from 18 to 77 years, while the age of mothers ranged between 17 and 50.

The mean age of the husband was 38.18 ± 9.78 and the mean age of the wife was 32.28 ± 7.80. Almost half of the participants, 105 (55.3%), were residing in rural areas.

The monthly income of 44 (23.2%) families was in the range of 5000–10,000, 56 (29.5%) had a family income of 10,000–20,000, and 90 (47.4%) earned above 20,000. The majority of mothers, 120 (63.2%), were illiterate, while the literacy rate among fathers was 119 (62.6%).

Among participants, 158 (83.2%) parents were married to their cousins, and only 32 (16.8%) couples didn't have any blood relations. The average number of children of these parents was 3.78 ± 1.88, and among these, thalassemia‐affected children were 1.30 ± 0.55, as described in Table 1.

**Table 1:** Socioeconomic data of the participants in this study, including housing, literacy rates, relationships, and age.

Variables	Frequency (%)
Address	
Rural	105 (55.3%)
Urban	85 (44.7%)
Father literacy	
Literate	119 (62.6%)
Illiterate	71 (37.4%)
Mother literacy	
Literate	70 (36.8%)
Illiterate	120 (63.2%)
Relation between couples	
Cousins	158 (83.2%)
Out of family	32 (16.8%)
Monthly income	
5000–10,000	44 (23.2%)
10,000–20,000	56 (29.5%)
More than 20,000	90 (47.4%)
Husband age	
18–29	31 (16.3%)
30–41	100 (52.6%)
42–53	47 (24.7%)
54–65	10 (5.3%)
66–77	2 (1.1%)
Wife age	
17–23	22 (11.6%)
24–30	74 (38.9%)
31–37	44 (23.2%)
38–44	29 (15.3%)
45–50	21 (11.1%)

### 3.2 Awareness Assessment

In the present study, out of 190, only 26 (13.7%) subjects knew that thalassemia is an inherited disorder. The majority of parents, 174 (91.6%), didn't know that this disease can be identified during pregnancy, and they were also unaware of premarital screening for thalassemia.

Regarding treatment options, 154 of 190 (81.1%) parents believed that regular blood transfusion is the only treatment available for this disease.

Out of 190 participants, 95 (50%) acknowledged that the incidence of thalassemia is increased by consanguineous marriages, while 50% believed thalassemia is not related to consanguineous marriages (Table 2).

**Table 2:** Questions were asked to survey participants regarding their awareness of thalassemia.

Questions about thalassemia	Aware	Not aware
Do you know thalassemia is an inherited disease?	26 (13.7%)	164 (86.3%)
Did you know thalassemia can be detected during pregnancy?	16 (8.4%)	174 (91.6%)
Did you know about screening tests for thalassemia before marriage?	16 (8.4%)	174 (91.6%)
Do you think regular blood transfusion is the only treatment?	154 (81.1%)	36 (18.9%)
Do you think consanguineous marriage has any role to play in the incidence of thalassemia?	95 (50%)	95 (50%)

### 3.3 Awareness Scoring and Categorization

Based on the questions answered correctly, we have categorized the respondents into scores 0‐1 (poor knowledge), 2‐3 (average knowledge), and 4‐5 (good knowledge). Out of 190 parents, 93 (48.9%) had poor knowledge and 9 (4.7%) had good knowledge (Table 3).

**Table 3:** Levels of awareness in participants regarding thalassemia and its distributions.

	Awareness		
Level	Score range	Frequency (*f*)	Percentage (%)
Poor	0–1	93	48.9
Average	2–3	88	46.3
Good	4–5	9	4.7

### 3.4 Linear Regression Analysis

A linear regression analysis was conducted to investigate the factors influencing the Awareness Score. The model included several predictors: Wife's Education Level, Address, Couple Relationship, Income, Husband's Age, Husband's Education Level, and Wife's Age. The results demonstrate a model fit, with an *R*² value of 0.067. This indicates that only 6.7% of the variation in the Awareness Score is explained by the predictors included in the model. Model summary is represented in Table 4.

**Table 4:** Model summary of linear regression analysis.

Model summary									
Model	*R*	*R* ^2^	Adjusted *R* ^2^	Std. error of the estimate	Change Statistics				
					*R* ^2^ change	*F* change	df1	df2	Sig. *F* change
1	0.259^a^	0.067	0.031	0.57657	0.067	1.874	7	182	0.076

^a^ Predictors: (Constant), Wife's_Education_Level, Address, Couple_Relation, Income, Husband's Age, Husband's education level, Wife's Age.

The overall model was tested for significance using ANOVA, with an *F*‐statistic of 1.874 and a corresponding *p* value of 0.076, as demonstrated in Table 5.

**Table 5:** ANOVA results for linear regression model predicting awareness score.

ANOVA^a^						
Model		Sum of squares	df	Mean square	*F*	Sig.
**1**	**Regression**	**4.360**	**7**	**0.623**	**1.874**	**0.076** b
	**Residual**	**60.503**	**182**	**0.332**		
	**Total**	**64.863**	**189**			

^a^ Dependent variable: Awareness score.

^b^ Predictors: (Constant) Wife's_Education_Level, Address, Couple_Relation, Income, Husband's Age, Husband's_Education_Level, Wife's Age.

The individual predictors in the model were assessed for their contributions to the awareness score in Table 6.

**Table 6:** Coefficients for linear regression analysis showing coefficients, *t*‐statistic, *p* value and 95% confidence interval.

Coefficients^a^								
Model		Unstandardized coefficients		Standardized coefficients	*t*	Sig.	95.0% confidence interval for B	
		*B*	Std. Error	Beta			Lower bound	Upper bound
1	(Constant)	2.276	0.302		7.541	0.000	1.681	2.872
	Husband's age	0.026	0.077	0.036	0.335	0.738	−0.127	0.178
	Wife's age	−0.032	0.056	−0.065	−0.575	0.566	−0.143	0.079
	Couple_Relation	−0.304	0.114	−0.195	−2.662	0.008	−0.529	−0.079
	Address	−0.105	0.087	−0.089	−1.204	0.230	−0.276	0.067
	Income	−0.191	0.089	−0.163	−2.155	0.032	−0.366	−0.016
	Husband's_Education_Level	0.056	0.032	0.143	1.786	0.076	−0.006	0.119
	Wife's_Education_Level	−0.020	0.037	−0.045	−0.543	0.588	−0.093	0.053

^a^ Dependent Variable: Awareness Score.

The analysis revealed that Husband's Age had a coefficient of 0.026, with an insignificant *p* value of 0.738, suggesting no notable effect on the Awareness Score. Similarly, Wife's Age showed a coefficient of −0.304, with a *p* value of 0.056.

In contrast, the Couple's Relationship (Consanguinity) demonstrated a statistically significant relationship with the Awareness Score (*B* = −0.191, *p* = 0.020).

Residency (Rural/Urban) also had a significant impact, with a coefficient of −0.195 and a *p* value of 0.029. However, Income showed a coefficient of −0.063 and an insignificant *p* value of 0.556.

Both the Husband's Education Level, with a coefficient of 0.056 and a *p* value of 0.761, and the Wife's Education Level, with a coefficient of −0.078 and a *p* value of 0.520, showed no significant effect.

## 4 Discussion

Thalassemia is an autosomal recessive genetic disorder where ⍺ or β chains of hemoglobin are absent or decreased in quantity or quality [1]. This study was directed to address thalassemia major awareness among parents of thalassemia‐affected children. Our study found that participants had insufficient knowledge about the disease. The level of awareness was dependent on the residency of participants. Awareness was also better in couples who had consanguineous marriages.

In this study, only a few (13.7%) subjects knew that thalassemia is an inherited disease, while in Malaysia, most of the parents (87.5%) knew thalassemia is transferred to children from parents, and blood tests can be identified easily [13]. Our results are quite similar to a study in India, where only 12.7% knew about the genetic transmission of the disease [14]. These differences may be attributed to variants in public health initiatives, education outreach programs, and the availability of genetic counseling services across different regions. Countries with established thalassemia awareness campaigns and screening programs tend to report higher knowledge levels among affected families.

Most of our study participants were unaware of premarital screening and detection of thalassemia during pregnancy. This result is in contrast to a study conducted on thalassemic parents in Swat, where most of the respondents (63.5%) know of thalassemia detection during pregnancy, and it can be helpful for the prevention of disease. Their population had good awareness because they were facilitated by genetic counseling and screening facilities that are not available in our community [10]. This result is comparable to a study that shows that 76.34% of parents did not have awareness about premarital screening [15], and this is dissimilar to a previous study by Kamran Ishfaq et al., in which 169 (56.3%) knew about premarital screening [9].

Our study revealed half of the parents know about the increasing incidence of thalassemia among cousin marriages, in contrast to our findings from a study conducted in Pakistan in which 83% were unaware that consanguineous marriage is associated with thalassemia [16]. Another study in India, where 73% were not aware of the risk of thalassemia major in cousin marriages [17]. The differences in awareness levels may stem from cultural and religious factors, as cousin marriages are more prevalent and socially accepted in certain communities. In regions where cousin marriages are a common tradition, awareness campaigns focusing on genetic risks may not be as widespread or effective.

The majority of parents (81.1%) think that thalassemia can be treated only by blood transfusion; similarly, 77.12% of participants in another study by Ankur Jain et al. believe that transfusion is the only treatment, while 6.78% think that bone marrow transplantation is the treatment of thalassemia [14], almost the same results reported in Bangladesh, where only 23.4% mentioned bone marrow transplant as a treatment option [18].

Regarding the level of awareness about thalassemia, the majority of parents (48.9%) had poor knowledge and only 4.7% were aware of the disease. Due to lower maternal education levels and rural parental residence, the current study reveals a poorer level of awareness compared to the study in Pakistan where 69% of respondents were aware, and only 31% have poor knowledge of the disease [4].

The result of this study found that couples' relationships (Consanguinity) have a statistically significant relationship with the Awareness Score (*B* = −0.191, *p* = 0.020). This may be because majority of them were aware of the increased probability of having a thalassemia child due to cousin marriages. 72% of respondents in Sahiwal Punjab agreed that cousin marriage is a major risk factor for thalassemia and had a fair knowledge of the basics of the disease [19]. Similarly, another study reported that 79% of subjects had cousin marriage, but when asked about their opinion about cousin marriage, 97% of participants recognized the increasing risk of genetic disorders and discouraged cousin marriage in their children [20].

Our study indicates that living areas (urban/rural) also have a significant impact on awareness and another study from Pakistan also reported that knowledge attitudes and practices regarding thalassemia are more positive in urban areas than rural [21]. Similar results were also reported in Bangladesh where thalassemia awareness in urban residents was nearly double that of participants who resided in semi‐urban or rural areas [22]. The other predictors, including Husband's Age, Wife's Age, Income, Husband's Education Level, and Wife's Education Level, were not found to influence the Awareness Score significantly.

Our study offers policymakers practical insights to develop effective, evidence‐based strategies for thalassemia prevention and management. It highlights the crucial role of female education in improving health outcomes, emphasizing the need for investments in programs promoting female literacy. The findings also inform policymakers on designing interventions that educate parents about consanguinity risks, promote premarital screening, and provide genetic counseling. This study underscores the importance of collaboration between healthcare providers, educators, community leaders, and policymakers to foster a holistic approach to thalassemia prevention. Furthermore, it emphasizes the need for public health campaigns to address knowledge gaps and raise awareness, ultimately reducing thalassemia incidence and improving the quality of life for affected families.

## 5 Conclusion

Our study concluded knowledge regarding thalassemia in parents of patients affected with thalassemia is not satisfactory, which further leads to failure of prevention and increasing incidence of disease. As we found in our study, the level of awareness is associated with residency, which indicates the dire need for interventional educational programs in rural areas to enhance awareness among the general population and especially in high‐risk families. Government and health care professionals should guide thalassemia‐affected families about premarital and antenatal screening to reduce the burden of disease in the country. To effectively address this issue in Balochistan, public health policies should focus on integrating thalassemia education into school curricula, community outreach programs, and mandatory premarital screening initiatives. Additionally, healthcare infrastructure must be strengthened to ensure accessible genetic counseling services and early diagnosis programs, particularly in rural and underserved areas.

## 6 Limitations

This study was conducted solely in one thalassemia center in Mekran, which was due to the unavailability of resources; furthermore, the study may have been conducted with a larger sample size, but despite multiple reminders and requests, some families didn't participate in the study. As we applied the convenient sampling technique, the findings may not be generalizable. We assessed the knowledge of parents about only a few aspects of thalassemia, limiting the idea of overall awareness of the study population about the disease. Additionally, response bias may have influenced the results, as parents who were more aware of thalassemia may have been more willing to participate. Furthermore, there is a possibility of misreporting, as some parents may have overestimated their knowledge due to social desirability bias or misunderstanding of the questions.

## Author Contributions

**Zarnum Gul:** conceptualization, methodology, writing – original draft, software, formal analysis, investigation, validation, writing – review and editing, data curation. **Mehtab Malik:** methodology, writing – original draft, software, data curation. **Uzzi Uzair:** conceptualization, methodology, software, writing – original draft, formal analysis, investigation, validation, writing – review and editing, data curation. **Aiman Baloch:** writing – review and editing, writing – original draft. **Quang Dai La:** writing – review and editing, writing – original draft. **Noman Sadiq:** supervision. **David F Lo:** supervision.

## Conflicts of Interest

The authors declare no conflicts of interest.

## Transparency Statement

The lead author David F. Lo affirms that this manuscript is an honest, accurate, and transparent account of the study being reported; that no important aspects of the study have been omitted; and that any discrepancies from the study as planned (and, if relevant, registered) have been explained.

## ERC Approval

This sampling in this study paper has received appropriate ERC approval.

## Data Availability

The data that support the findings of this study are available on request from the corresponding author. The data are not publicly available due to privacy or ethical restrictions.
